# SRSF3 and hnRNP A1-mediated m6A-modified circCDK14 regulates intramuscular fat deposition by acting as miR-4492-z sponge

**DOI:** 10.1186/s11658-025-00699-6

**Published:** 2025-03-04

**Authors:** Chunyu Qin, Fang Xu, Binglin Yue, Jincheng Zhong, Zhixin Chai, Hui Wang

**Affiliations:** Key Laboratory of Qinghai-Tibetan Plateau Animal Genetic Resource Reservation and Utilization, Sichuan Province and Ministry of Education, Southwest Minzu University, Chengdu, 610225 China

**Keywords:** circCDK14, m6A, SRSF3, hnRNP A1, Cell proliferation and differentiation

## Abstract

The intramuscular fat (IMF) content of yak beef is critical for determining its quality. Circular RNAs (circRNAs) are a group of endogenous non-coding RNAs that have emerged as important factors in the regulation of IMF deposition. However, the molecular mechanisms through which circRNAs regulate IMF deposition, particularly in yaks, remain unclear. In the present study, a novel circRNA, circCDK14 (originating from the yak’s *CDK14* gene), was identified by sequencing and RNase R treatment. In our previous study, we successfully established a ceRNA network map and identified miR-4492-z, which interacts with circCDK14. Furthermore, using methylation prediction software, we predicted two genes, *SRSF3* and *hnRNP A1*, that have a strong binding relationship with circCDK14; existing research has confirmed their close association with m6A methylation modifications. On the basis of these findings, we comprehensively evaluated the effects of circCDK14, miR-4492-z, SRSF3 and hnRNP A1 on the proliferation and differentiation of yak intramuscular pre-adipocytes using EdU, CCK-8, BODIPY, Oil Red O and qRT-PCR analyses. Mechanistically, the interaction between circCDK14 and miR-4492-z was validated using a dual-luciferase reporter gene assay and rescue experiments. RIP assays revealed the binding interaction of circCDK14 with SRSF3 and hnRNP A1. The MeRIP experiments showed modification of circCDK14 methylation, with SRSF3 and hnRNP A1 promoting the methylation and translocation of circCDK14 from the nucleus to the cytoplasm. In summary, our results suggest that m6A-modified circCDK14 plays a crucial role as an miR-4492-z sponge in regulating IMF deposition in yaks and that the nuclear export of circCDK14 correlates with the expression levels of SRSF3 and hnRNP A1. This study provides a theoretical basis for the improvement of yak meat quality and promotes the development of molecular yak breeding.

**Supplementary Information:**

The online version contains supplementary material available at 10.1186/s11658-025-00699-6.

## Introduction

Yaks (*Bos grunniens*) are multipurpose animals adapted to cold and oxygen-deprived environments that provide milk, meat, wool and transportation to the indigenous people of the Qinghai-Tibetan Plateau [1]. Adipose tissue serves as a crucial endocrine organ in yaks that allows them to adapt to the alpine environment. It is classified into three different types according to its anatomical location, morphology and function: white adipose tissue (WAT), brown adipose tissue and beige adipose tissue [2]. Intramuscular fat (IMF) is a form of WAT that significantly influences beef grades and enhances meat quality. The IMF accumulation is primarily influenced by differentiation of intramuscular pre-adipocytes and lipid storage in mature adipocytes [3]. However, the low IMF content of yak meat results in reduced tenderness, flavour and juiciness, thereby limiting its market appeal. Therefore, it is imperative to study the mechanisms governing the proliferation and differentiation of yak intramuscular pre-adipocytes (YIMAs) to enhance meat production efficiency and quality.

Circular RNAs (circRNAs) are endogenous non-coding RNAs with covalently closed structures at the 5′ and 3′ ends formed by reverse splicing of precursor mRNAs in the nucleus, which are different from miRNAs and long-stranded non-coding RNAs (lncRNAs) [4, 5]. CircRNAs exhibit enhanced stability compared with their linear transcripts owing to cell/tissue-specific and stage-specific expression, as well as unique molecular structures [6, 7]. Numerous studies have demonstrated the role of circRNAs in neurodevelopment [8, 9], muscle development [10, 11], adipogenesis [12, 13] and other physiological processes. Su et al. [14] and Qin et al. [14] analysed the profiling of circRNAs in the yak IMF. However, only Qin et al. [14] have reported that circCWC22 regulates yak IMF deposition by acting as an miR-3059-x sponge to further regulate the expression of *HMGCL*. Therefore, the functions of circRNAs in yak adipogenesis and their regulatory mechanisms require further exploration.

The localisation of circRNAs determines their mechanisms of action. CircRNAs in the nucleus regulate transcription and splicing, whereas those in the cytoplasm function as competitive endogenous RNAs (ceRNAs) that act as miRNA ‘sponges’. To function in the cytoplasm, circRNAs must be transported from the nucleus via specific mechanisms potentially involving RNA-binding proteins, N6-methyladenosine (m6A) and other RNA export mechanisms [15, 16]. The m6A modification, the most prevalent RNA modification in eukaryotes, plays a crucial role in circRNA biogenesis [16], translation [17] and cytoplasmic export [18]. These modifications are influenced by the m6A ‘writer’, ‘eraser’ and ‘reader’ complexes [19]. Further exploration of the regulatory factors affecting m6A modification of circRNAs is essential for a comprehensive understanding of circRNA functionality.

Serine/arginine-rich splicing factor 3 (SRSF3) is a member of the highly conserved SR protein family. It plays a pivotal role in the regulation of RNA-selective splicing, RNA export and other crucial biological processes [20]. Recent studies have highlighted the significance of SRSF3 as a key regulator of m6A modifications in both lncRNAs and mRNA [21, 22]. However, the relationship between SRSF3 and m6A-modified circRNAs must be explored in depth to clarify their synergistic roles in various biological processes. Nuclear ribonucleoprotein A1 (hnRNP A1) is involved in various metabolic processes, including RNA splicing, nucleation, stabilisation and translation [23], and is particularly important in maintaining the homeostatic regulation of liver [24], muscle [25] and lipid metabolism [26]. In addition, the interaction between SRSF3 and hnRNP A1 is a key aspect of splicing regulation [27] and has implications for tumourigenesis and development [28]. This study extensively explored the roles of SRSF3 and hnRNP A1 in YIMAs and revealed their regulatory effects on m6A modification of circRNAs.

This study investigated the relationship between SRSF3, hnRNP A1, m6A modifications and circRNA nuclear export to improve our understanding of how circCDK14 affects yak IMF. These findings provide novel insights into the molecular mechanisms by which epigenetic modifications of RNA regulate the proliferation and differentiation of YIMAs.

## Materials and methods

### Sample collection

Yak longissimus dorsi muscle tissue samples were collected from Xiaojin County, Aba Tibetan and Qiang Autonomous Prefecture, Sichuan Province, China. Comparable-weight yaks were raised on natural pasture until they reached 4 years of age, following a grazing system that ensured uniform treatment and nutritional conditions. Subsequently, they were fattened for 6 months under identical nutritional conditions. All yaks were euthanised according to humanitarian principles to reduce animal suffering. The longissimus dorsi muscle tissue of the yak was rapidly collected after euthanasia to ensure freshness of the sample.

### Isolation and culture of YIMAs

Adipose tissue was extracted from the longissimus dorsi muscle of yaks and rinsed repeatedly with phosphate-buffered saline (PBS) solution containing 2–5% penicillin–streptomycin (BasalMedia, Shanghai, China). Under strict aseptic conditions, the tissue was minced and digested with 0.1% collagenase I and II (Thermo Fisher Scientific, Waltham, MA, USA) for a duration of 3 h. Digestion was terminated by adding an equal volume of 10% foetal bovine serum (FBS; Gibco, Waltham, MA, USA). The resulting digested mixture was filtered, and the cell pellet was collected by differential centrifugation at 2000, 1800 and 1500 rpm. The cells were cultured in DMEM/F-12 (Gibco) supplemented with 10% FBS and 1% penicillin–streptomycin at 37 ℃ in a 5% CO_2_ incubator. Following inoculation, the culture was incubated for 1.5 h, after which the medium was changed to remove non-adherent cells and debris, and attached cells were cultured [29]. An additional 100 µM oleic acid (Sigma-Aldrich, St Louis, MO, USA) was added as the inducing medium.

### Plasmid construction, small RNA synthesis and cell transfection

The complete sequence of circCDK14 and hnRNP A1 was integrated into the pCD25-ciR and pcDNA3.1 (+) vectors, respectively, to construct the pCD25-circ09813 and pcDNA3.1-hnRNP A1 overexpression plasmids, respectively. Moreover, the complete sequences of METTL3 and its mutations (D395A and W398A) were integrated into the pcDNA3.1 (+) vectors to create the pcDNA3.1-METTL3-WT and METTL3-MUT overexpression plasmids. The sequences of novel_circ_009813 (circCDK14) and *FBXO17* partial 3′ UTR, which are inversely complementary to miR-4492-z, were predicted using the BiBiServ server (https://bibiserv.cebitec.uni-bielefeld.de/rnahybrid) and then inserted into dual-luciferase reporter plasmid vectors (pGL3-Basic) to generate circCDK14 and *FBXO17* wild-type and mutant-type dual-luciferase reporter plasmids. The siRNAs and negative controls for circCDK14, SRSF3 and hnRNP A1 were synthesised by Tsingke Biotech (Beijing, China). The mimic and inhibitor of miR-4492-z were synthesised by GenePharma (Shanghai, China) (Supplementary Table S1). Cells were transfected using Lipofectamine 3000 (Invitrogen, Carlsbad, CA, USA) according to the manufacturer’s protocol at a density of 70–80% [30].

### Total RNA extraction, reverse transcription and quantitative real-time reverse transcription PCR (qRT-PCR)

Trizol reagent (Takara, Kusatsu, Japan) was used to extract total RNA, and the RNA quality was evaluated to ensure that the OD260/OD280 ratio was in the appropriate range of 1.8 to 2.0. Subsequently, RNA was reverse-transcribed into cDNA using a PrimeScript RT Reagent Kit (Takara). For RNase R treatment, the isolated RNA underwent incubation with RNase R (Geneseed, Guangzhou, China) for 3 min at 72 ℃ and 30 min at 37 ℃, with a control group treated without RNase R. Special reverse transcription primers were used for miR-4492-z and *U6*. The relative expression levels of circRNAs, mRNAs and miRNAs were quantified using a SYBR Premix Ex Taq kit (Takara). The 2^*−ΔΔCt*^ method was employed with *GAPDH* and *U6* as internal reference genes. Tsingke Biotech synthesised all the RNA primers (Supplementary Table S2). In this study, we primarily examined genes associated with cell proliferation, including *cyclin B*, *cyclin D*, *cyclin E* and *KI67*, as well as genes related to adipocyte differentiation, such as *PPARγ*, *C/EBPα*, *FASN* and *SREBP1*.

### Nuclear/cytoplasmic separation

RNA isolation of nuclear and cytoplasmic fractions was performed using the PARIS™ Kit (Thermo Fisher Scientific) following the manufacturer’s protocols [31, 32]. The isolated RNA was then reverse transcribed and amplified using qRT-PCR as described above, using *U6* as the nuclear control and *GAPDH* as the cytoplasmic control.

### Oil red O and BODIPY staining

Differentiated cells were fixed using 4% paraformaldehyde (Biosharp, Hefei, China) for 30 min at ambient temperature, followed by washing with PBS (BasalMedia) and staining with either a freshly prepared 60% Oil Red O solution (Merck/Millipore, Burlington, MA, USA) or 1 μg/mL BODIPY solution (Thermo Fisher Scientific) for 30 min. Subsequently, the cells were rinsed with PBS and nuclei were stained with DAPI (Solarbio, Beijing, China) for 5 min in the dark at 37 ℃. After rinsing with PBS, lipid-forming staining was visualised using fluorescence microscopy (Carl Zeiss, Germany). Isopropanol (Chron Chemicals, Chengdu, China) was added to extract the Oil Red O staining solution, and the absorbance value of the mixture was measured at 510 nm using a microplate reader (Thermo Fisher Scientific).

### Cell counting kit 8 (CCK-8) and 5-ethynyl-2′-deoxyuridine (EdU) assays

Cell proliferation was evaluated using CCK-8 (Oriscience, Chengdu, China) and EdU (Beyotime, Shanghai, China). At 0, 12, 24 and 48 h post-transfection, 10 μL CCK-8 solution was added to each well of a 96-well plate. The plate was then incubated in the dark for 1 h, followed by the measurement of absorbance at 450 nm using an enzyme marker. Additionally, 48 h post-transfection, cells in 12-well plates were treated with 1 × EdU stock solution at 37 ℃ for 2 h. Subsequently, the cells were fixed with 4% paraformaldehyde for 30 min at room temperature, stained according to the manufacturer’s protocols [32] and observed using fluorescence microscopy.

### Dual-luciferase reporter assay

The YIMAs were cultured in 24-well plates and co-transfected with 250 ng dual-luciferase reporter plasmid vectors, 10 ng TK and 180 nM miRNA mimic or control using Lipofectamine 3000. Luciferase activity was assessed 48 h post-treatment using a dual-luciferase reporter assay kit (Promega, Madison, WI, USA) [33]. Relative luciferase activity was determined by calculating the ratio of firefly to Renilla luciferase activity.

### RNA immunoprecipitation (RIP)

The interaction between SRSF3, hnRNP A1 and circCDK14 was identified using an RIP kit (BersinBio, Guangzhou, China) following the manufacturer’s protocol [34]. The enrichment rate of circCDK14 associated with each antibody was assessed using qRT-PCR. The SRSF3 antibody was procured from Santa Cruz Biotechnology (Dallas, TX, USA), hnRNP A1 was purchased from ABclonal Technology (Wuhan, China) and METTL3 was purchased from Proteintech Biotechnology (Wuhan, China).

### Methylated RNA immunoprecipitation (MeRIP)

Quantification of m6A-modified circCDK14 was performed using the MeRIP kit (BersinBio) according to the manufacturer’s protocol [35]. The enrichment rate of circCDK14 was verified using real-time quantitative reverse transcription polymerase chain reaction (qRT-PCR).

### Western blot

Total cellular proteins were harvested 72 h post-transfection using radioimmunoprecipitation assay lysis buffer (Beyotime). A BCA protein assay kit (Thermo Fisher Scientific) was used to determine the protein concentration in each sample. Proteins were separated using 10% sodium dodecyl sulphate–polyacrylamide gel electrophoresis (Beyotime), transferred to a polyvinylidene fluoride membrane (Millipore) and blocked with 5% milk at room temperature for 2 h. Membranes were incubated with primary antibodies for SRSF3 (1:1000, Santa Cruz Biotechnology), hnRNP A1 (1:1000, ABclonal Technology) and β-actin (1:5000, Proteintech, Wuhan, China) for 12 h at 4 ℃, followed by horseradish peroxidase (HRP) goat anti-rabbit immunoglobulin (Ig)G (H + L) (1:5000, ABclonal Technology) and HRP-conjugated Affinipure goat anti-mouse IgG (H + L) (1:5000, Proteintech) secondary antibodies for 1.5 h at room temperature. Enhanced chemiluminescence (Biosharp) was used to visualise the staining. Protein bands were imaged using a Gel Doc XR + Imager (Bio-Rad, Hercules, CA, USA).

### Statistical analysis

The differences between groups were tested using Student’s *t*-test and analysis of variance (ANOVA), with all data expressed as mean ± standard error of the mean (SEM). Bar and line graphs were generated using GraphPad 8.0 (GraphPad Software, La Jolla, CA, USA). Significance was set at* P* < 0.05 with the following abbreviations: *P < 0.05, **P < 0.01, ***P < 0.001 and ns = not significant.

## Results

### Characterisation of circCDK14 in YIMAs

**Fig. 1:**
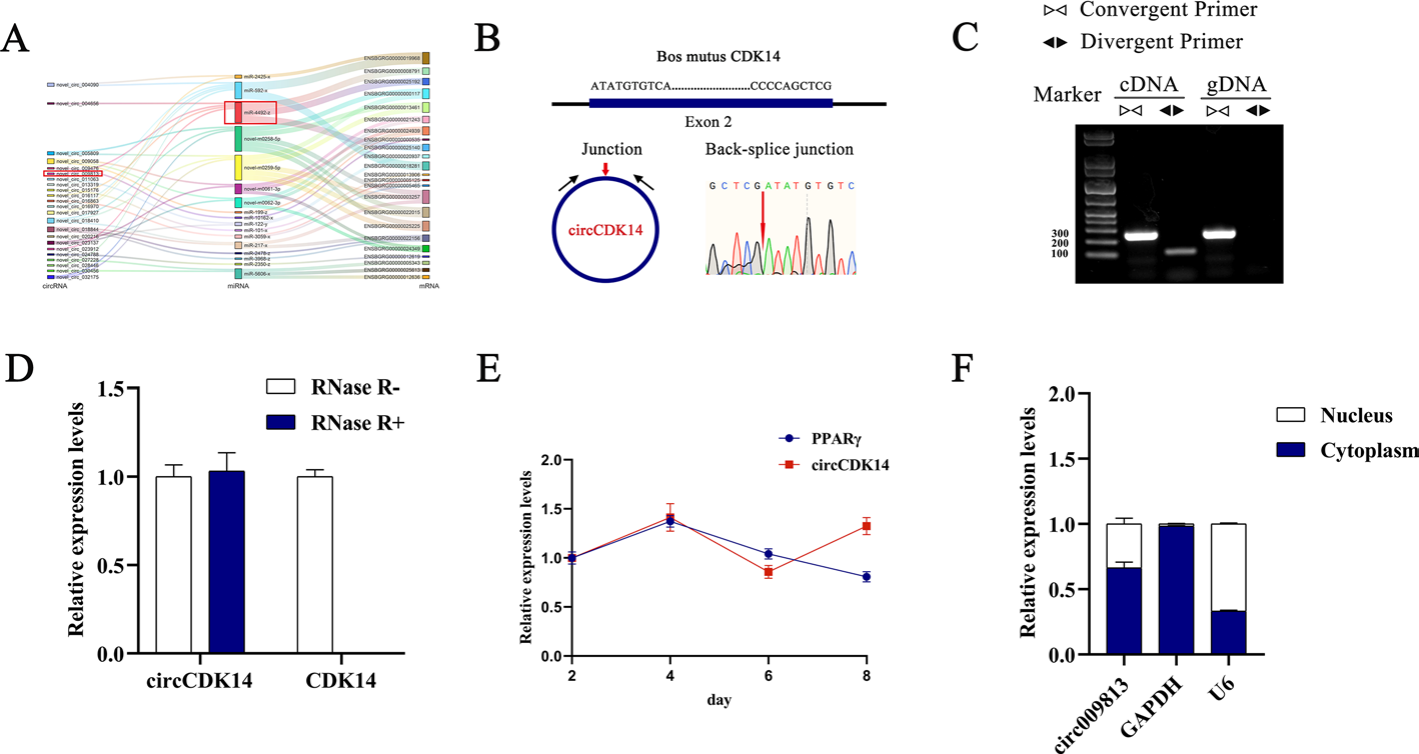
The identification of circCDK14 in YIMAs. **A** Prediction of the circCDK14-miR-4492-z axis; **B** schematic representation of the *CDK14* gene producing circCDK14; **C** detection of circCDK14 and *CDK14* in cDNA and gDNA using divergent and convergent primers; **D** expressions of circCDK14 and *CDK14* were assessed after treatment with RNase R; **E** dynamics of circCDK14 expression during the differentiation of YIMAs; **F** circCWC22 expression in the nucleus and cytoplasm. All experiments were performed in triplicate and repeated 3 times (*n* = 9)

In our previous study, we observed a significant difference in the expression of circCDK14 between high- and low-IMF tissues, which warranted further investigation [14] (Fig. 1A). This circRNA, derived from the second exon of *CDK14* and named circCDK14, has a length of 249 nt (Fig. 1B). To verify its circular structure, amplification was performed using divergent and convergent primers (Fig. 1C). qRT-PCR analysis of total RNA from YIMAs treated with RNase R indicated that circCDK14 was more stable than *CDK14* (Fig. 1D). The expression patterns of circCDK14 and *PPARγ* remained consistent during the 6-day period following differentiation of YIMAs (Fig. 1E). Nucleoplasmic separation assays revealed that circCDK14 was mainly localised to the cytoplasm (Fig. 1F), suggesting its potential role as a ceRNA.

### CircCDK14 promotes proliferation and inhibits differentiation of YIMAs

**Fig. 2:**
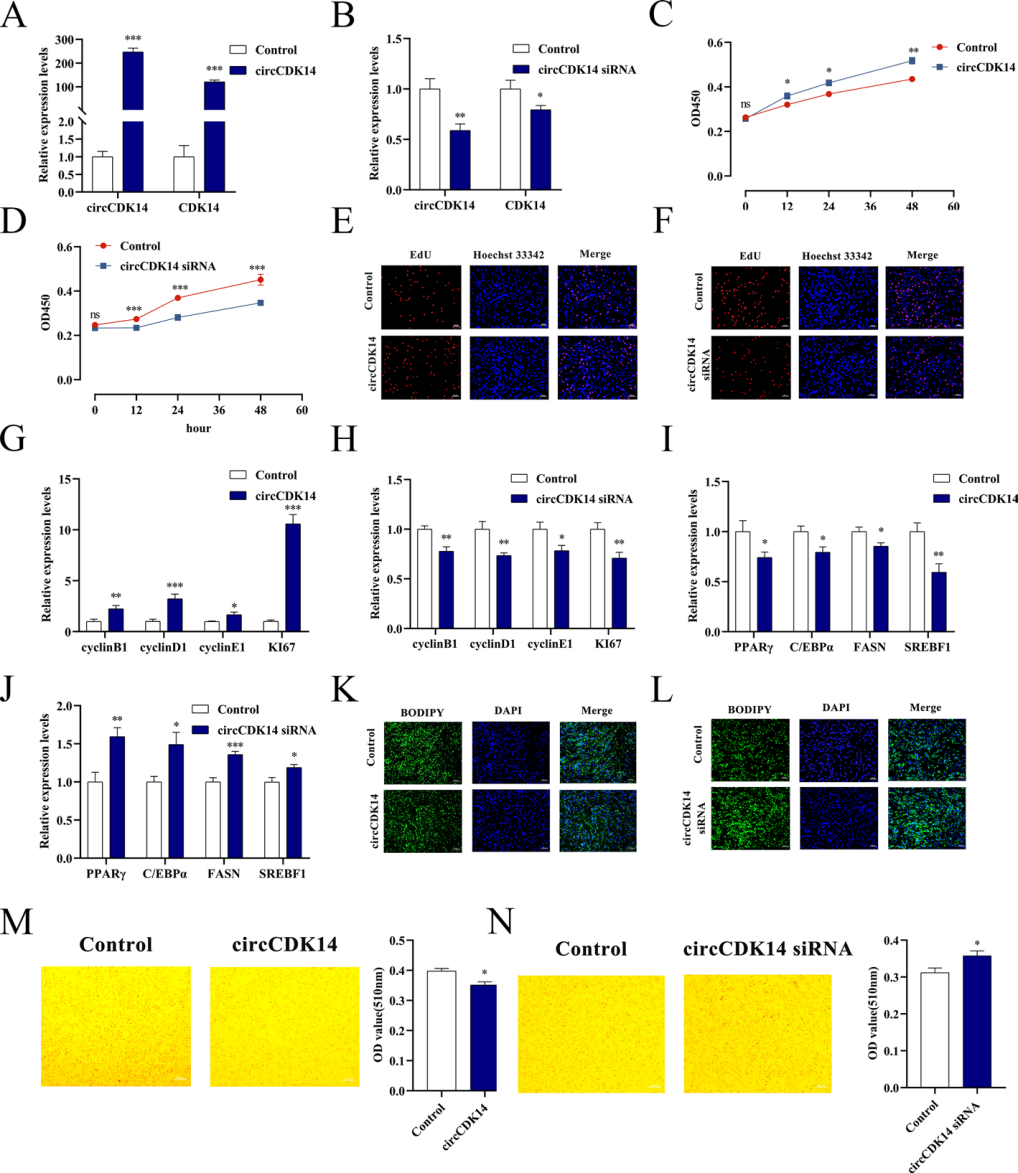
The impact of circCDK14 on YIMAs’ proliferation and differentiation. **A**–**B** The level of circCDK14 overexpression or interference; **C**–**D** CCK-8 assay after circCDK14 overexpression or interference; **E**–**F** EdU staining post- circCDK14 overexpression or interference; **G**–**H** proliferation marker gene expression post-circCDK14 overexpression or interference; **I**–**J** differentiation marker gene expression post-circCDK14 overexpression or interference; **K**–**L** bodipy staining post-circCDK14 overexpression or interference; **M**–**N** Oil Red O staining post-circCDK14 overexpression or interference. The CCK-8 assay was repeated 6 times (*n* = 6), and the other experiments were performed in triplicate and repeated 3 times (*n* = 9)

A series of in vitro experiments was conducted to investigate the biological functions of circCDK14. The qRT-PCR results indicated that the overexpression of circCDK14 led to a significant increase in its expression level compared with control cells, whereas circCDK14 siRNA effectively reduced its expression (Fig. 2A, B). CCK-8 and EdU assays revealed that overexpression of circCDK14 significantly increased the proliferation YIMAs compared with control cells, whereas its knockdown substantially suppressed this effect (Fig. 2C–F). Furthermore, qRT-PCR results revealed that overexpression of circCDK14 promoted the mRNA expression levels of genes associated with cell proliferation compared with that of the control, whereas downregulation of circCDK14 significantly inhibited their expression (Fig. 2G, H). Moreover, circCDK14 overexpression in YIMAs led to a decrease in the mRNA expression of differentiation markers compared with that in the control. Conversely, downregulation of circCDK14 showed the opposite trend (Fig. 2I, J). In addition, BODIPY and Oil Red O assays showed that overexpression of circCDK14 reduced the formation of lipid droplets compared with control cells, whereas downregulation of circCDK14 promoted their accumulation (Fig. 2K–N). These results indicate that circCDK14 contributes to the enhancement of YIMA proliferation and the suppression of cell differentiation.

### CircCDK14 regulates IMF deposition in yak by targeting miR-4492-z

**Fig. 3:**
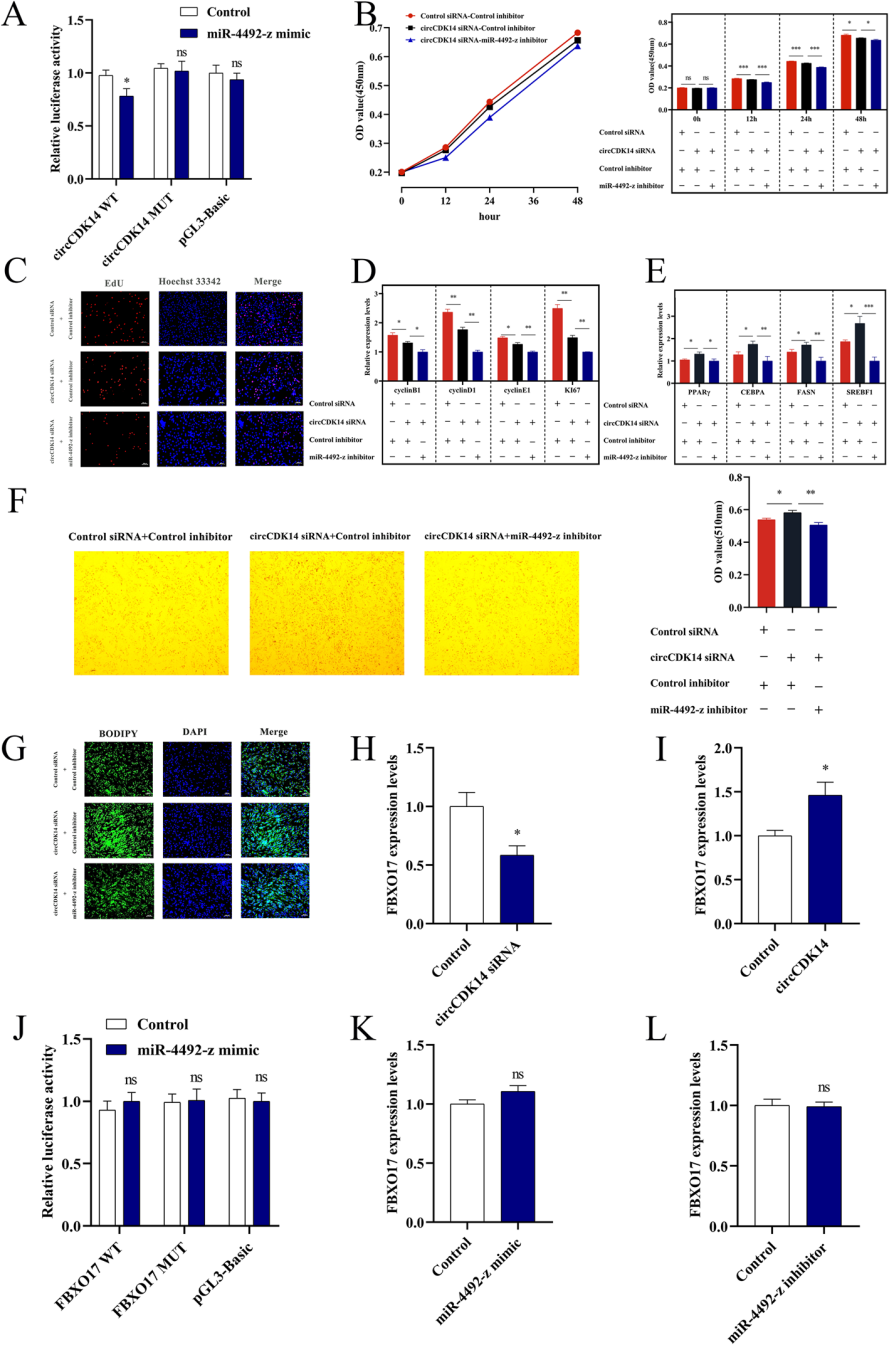
CircCDK14 regulates yak IMF deposition through miR-4492-z. **A** Dual-luciferase assay to detect miR-4492-z binding to circCDK14; co-transfection with circCDK14 siRNA versus a control siRNA and either the miR-4492-z inhibitor or a control, **B** CCK-8 assay; **C** EdU staining; **D** expression level of proliferation marker genes; **E** expression levels of differentiation marker genes; **F** Oil Red O staining; **G** BODIPY staining; **H**–**I** expression of *FBXO17* after interference or overexpression with circCDK14; **J** dual-luciferase assay for detection of miR-4492-z binding to *FBXO17*; **K**–**L** expression of *FBXO17* after overexpression or interference of miR-4492-z. The CCK-8 assay was repeated 6 times (*n* = 6), and the other experiments were performed in triplicate and repeated 3 times (*n* = 9)

Our previous bioinformatics analysis suggested that there is a potential targeting relationship among circCDK14, *FBXO17* and miR-4492-z (Fig. 1A) [14]. Dual-luciferase assays showed that the luciferase activity of wild-type circCDK14 was decreased by the miR-4492-z mimic compared with control cells, with no significant change observed in the mutant group (Fig. 3A), indicating a specific interaction between circCDK14 and miR-4492-z. Co-transfection of circCDK14 siRNA and the miR-4492-z inhibitor in YIMAs was performed to assess whether the miR-4492-z inhibitor counteracted the effect of circCDK14 siRNA on yak IMF deposition. CCK-8 and EdU assays demonstrated that the inhibition of miR-4492-z amplified the inhibitory effect of circCDK14 siRNA on the proliferation of YIMAs (Fig. 3B, C). qRT-PCR analysis further demonstrated that the miR-4492-z inhibitor facilitated a reduction in the expression of proliferation marker genes caused by circCDK14 siRNA (Fig. 3D). Furthermore, the miR-4492-z inhibitor partially restored the promoting effect of circCDK14 siRNA on the expression of cell differentiation marker genes in YIMAs (Fig. 3E). BODIPY and Oil Red O staining revealed that the miR-4492-z inhibitor partially counteracted the promotion of IMF deposition by circCDK14 siRNA (Fig. 3F, G). Collectively, these results suggest that circCDK14 modulates the proliferation and differentiation of YIMAs via miR-4492-z.

Knockdown of circCDK14 decreased *FBXO17* expression compared with control cells, whereas circCDK14 overexpression increased *FBXO17* expression (Fig. 3H, I). These findings indicated that circCDK14 may regulate *FBXO17* expression. However, the miR-4492-z mimic had no significant effect on the relative luminescence of the wild-type *FBXO17* compared with control cells (Fig. 3J), and miR-4492-z did not affect *FBXO17* expression (Fig. 3K, L). This finding suggests that there may be a direct or indirect regulatory relationship between circCDK14 and FBXO17 independent of miR-4492-z. The molecular mechanism by which circCDK14 regulates FBXO17 expression warrants further exploration.

### miR-4492-z promotes proliferation and differentiation of YIMAs

**Fig. 4:**
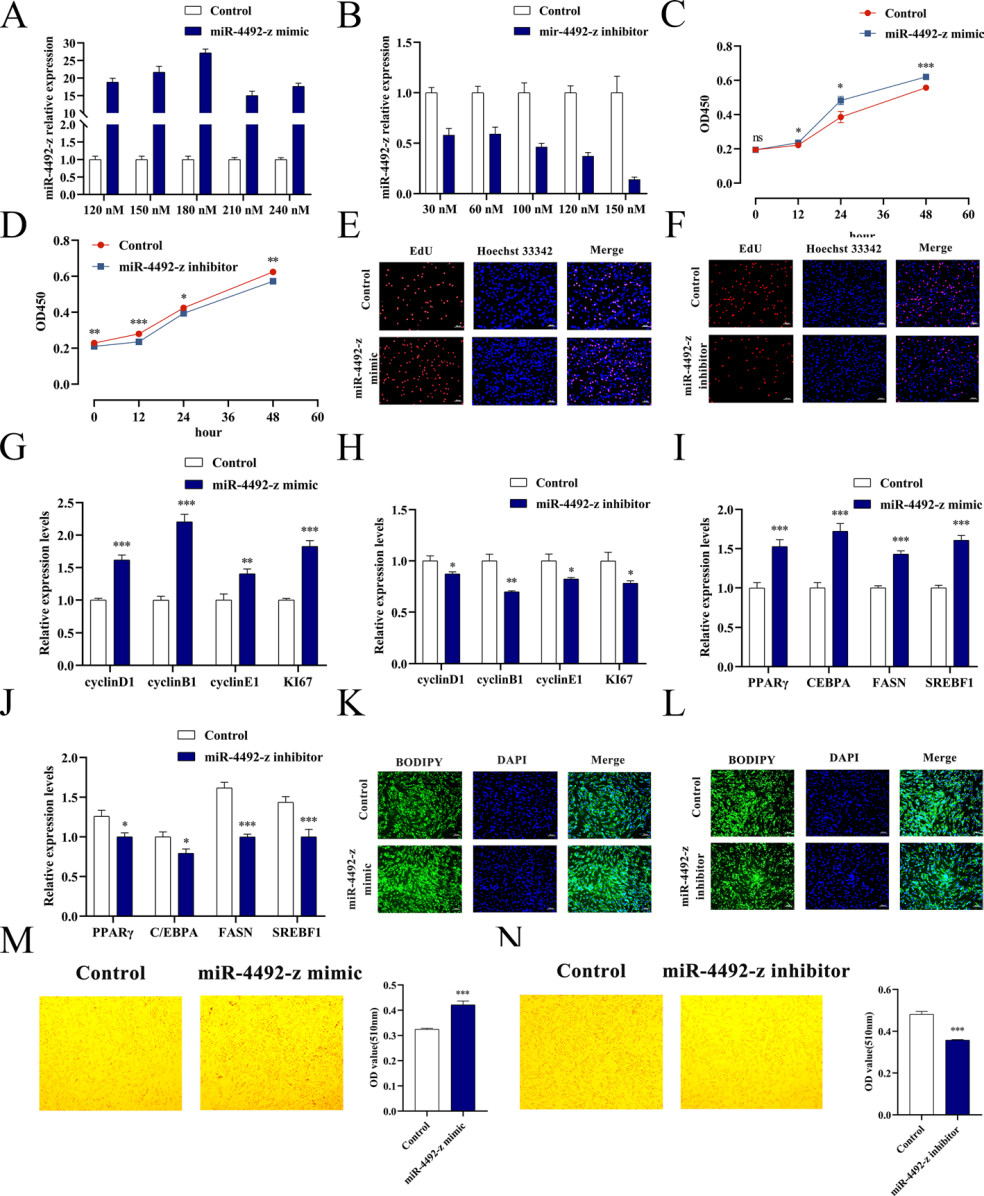
The impact of miR-4492-z on YIMAs’ proliferation and differentiation. **A** Overexpression level of miR-4492-z; **B** interference efficiency of miR-4492-z; **C**–**D** CCK-8 assay aftermiR-4492-z overexpression or interference; **E**–**F** EdU staining after miR-4492-z overexpression or interference; **G**–**H** proliferation marker gene expression after miR-4492-z overexpression or interference; **I**–**J** differentiation marker gene expression after miR-4492-z overexpression or interference; **K**–**L** BODIPY staining after miR-4492-z overexpression or interference; **M**–**N** Oil Red O staining after miR-4492-z overexpression. The CCK-8 assay was repeated 6 times (*n* = 6), and the other experiments were performed in triplicate and repeated 3 times (*n* = 9)

In the present study, we explored the role of miR-4492-z in YIMAs. After the transfection of the miR-4492-z mimic (180 nM) into YIMAs, qRT-PCR results showed significant overexpression of miR-4492-z compared with control cells (Fig. 4A). Conversely, transfection with the miR-4492-z inhibitor (150 nM) significantly inhibited miR-4492-z expression compared with that in the control (Fig. 4B). CCK-8 and EdU assays indicated that the miR-4492-z mimic promoted the proliferation of YIMAs compared with control cells, which was inhibited by the miR-4492-z inhibitor (Fig. 4C–F). Consistent results were observed in qRT-PCR analysis of proliferation marker genes (Fig. 4G, H). Moreover, the miR-4492-z mimic enhanced the mRNA expression of differentiation marker genes compared with control cells, whereas the miR-4492-z inhibitor had an inhibitory effect (Fig. 4I, J). BODIPY and Oil Red O staining corroborated these findings (Fig. 4K–N). Overall, these results suggest that miR-4492-z plays an important role in promoting the proliferation and differentiation of YIMAs.

### SRSF3 promotes nuclear export of circCDK14 through m6A modification

**Fig. 5:**
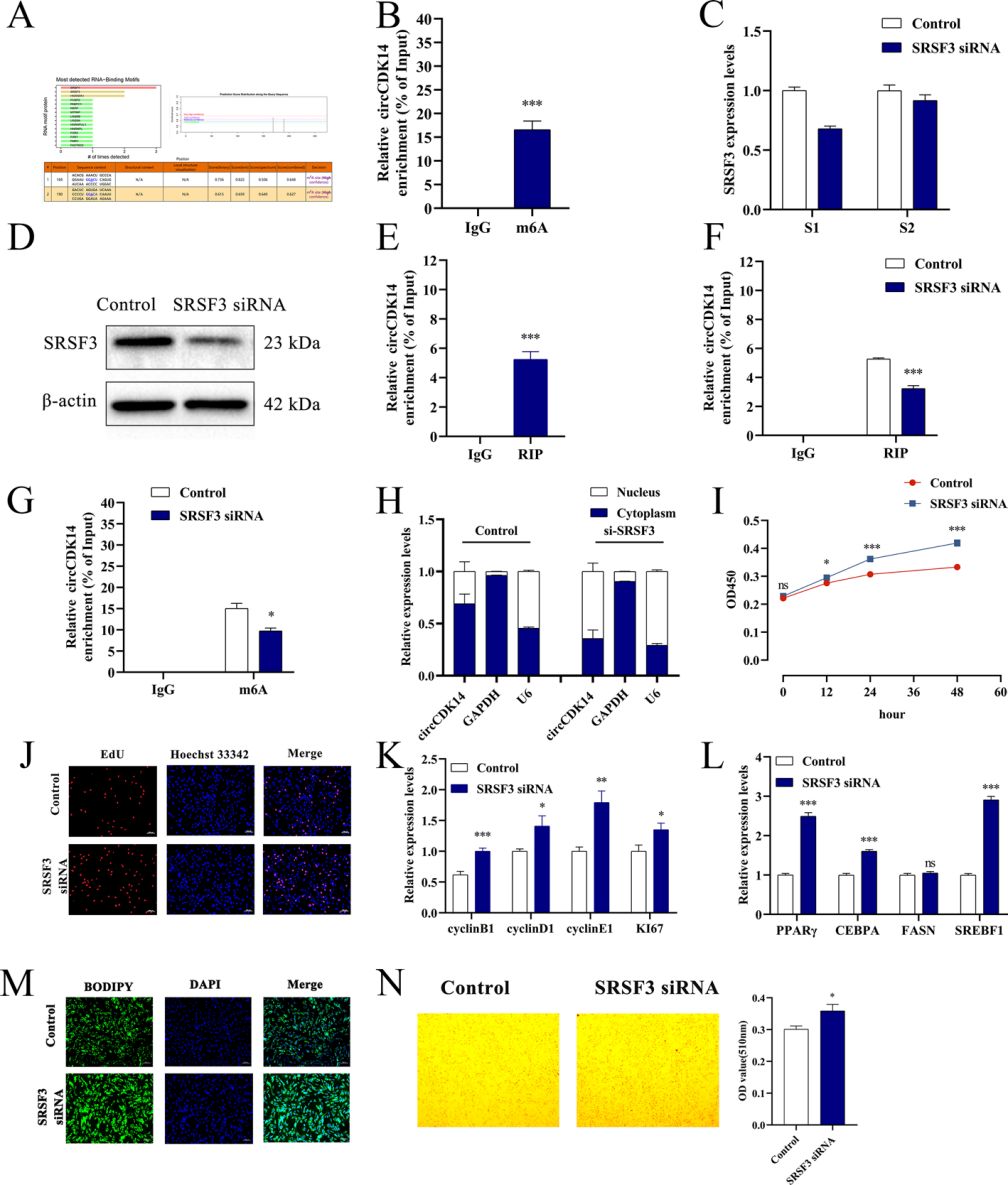
SRSF3 promotes nuclear export of m6A-modified circCDK14. **A** Prediction of RBPs and potential m6A sites of circCDK14; **B** MeRIP showing m6A antibody enrichment for circCDK14; *C * and *D* the SRSF3 mRNA (**C**) and protein (**D**) expression level after SRSF3 interference; **E** SRSF3 antibody for circCDK14 enrichment; after SRSF3 interference, **F** circCDK14 enrichment by SRSF3 antibody; **G** circCDK14 enrichment by m6A antibody; **H** circCDK14 expression in the nucleus and cytoplasm; **I** CCK-8 assay; **J** EdU staining; **K** expression levels of differentiation marker genes; **L** expression level of proliferation marker genes; **M** BODIPY staining; **N** Oil Red O staining. The western blot, RIP and MeRIP were repeated 3 times (*n* = 3), CCK-8 assay was repeated 6 times (*n* = 6) and other experiments were performed in triplicate and repeated 3 times (*n* = 9)

To further investigate the molecular mechanisms by which circCDK14 regulates the proliferation and differentiation of YIMAs, we used catRAPID (http://s.tartaglialab.com/page/catrapid_group) to predict the interactions between RNA-binding proteins (RBPs) and circCDK14. The results showed a potential interaction between circCDK14 and RBPs, including SRSF1, SRSF3 and hnRNP A1 (Fig. 5A). Notably, SRSF3, known for its role in RNA splicing, has also been reported to interact with m6A-modified RNA molecules [22]. This led us to hypothesise that there is an association between circCDK14 and m6A methylation. Using SRAMP (http://www.cuilab.cn/sramp) analysis, we identified two potential m6A sites in circCDK14, one of which was near its splice site (Fig. 5A). Subsequent MeRIP analysis confirmed the significant enrichment of circCDK14 in the m6A antibody immunoprecipitation complexes, validating the presence of m6A modifications (Fig. 5B). In addition, we designed two SRSF3 siRNAs, of which siRNA1 showed significant interference efficiency and was used for follow-up experiments (Fig. 5C, D). RIP revealed a binding interaction between SRSF3 and circCDK14, which was reduced by decreased SRSF3 expression (Fig. 5E, F). The MeRIP results revealed that downregulation of SRSF3 resulted in a reduction in the m6A level of circCDK14 compared with control cells (Fig. 5G). Subsequent nucleoplasmic separation assays demonstrated the predominant localisation of circCDK14 was nuclear rather than cytoplasmic following SRSF3 interference (Fig. 5H).

On the basis of the molecular interactions between SRSF3 and METTL3, we conducted a comprehensive investigation into whether the subcellular localization of circCDK14 is regulated by m6A modification. We confirmed the interaction between METTL3, SRSF3 and circCDK14 through RIP experiments (Supplementary Fig. 1A–H). Subsequently, we observed a decrease in the m6A methylation level of circCDK14 following effective inhibition of METTL3 (Supplementary Fig. 1I). In contrast, the overexpression of METTL3 resulted in the opposite effect (Supplementary Fig. 1J). Further experiments revealed that only the overexpression of wild-type METTL3 (OE-WT), and not the m6A catalytically defective METTL3 (OE-MUT), was able to restore the m6A methylation level of circCDK14 and the impaired transport from the nucleus to the cytoplasm after METTL3 disruption (Supplementary Fig. 1K, L). In addition, we found that the overexpression of METTL3 reinstated the m6A methylation level of circCDK14 and its transport from the nucleus to the cytoplasm, even under conditions where SRSF3 was disrupted, compared with controls (Supplementary Fig. 1M, N). These findings support the role of SRSF3 in binding to circCDK14 and facilitating its nuclear export to the cytoplasm in an m6A-dependent manner, a process that involves METTL3.

Next, we examined the effects of SRSF3 on fat deposition in yaks. CCK-8 and EdU assays revealed a significant increase in cell proliferation capacity and EdU incorporation following knockdown of SRSF3 compared with control cells (Fig. 5I, J). Furthermore, SRSF3 knockdown enhanced the expression of proliferation marker genes compared with control cells (Fig. 5K). Moreover, reduced SRSF3 expression resulted in increased mRNA expression of marker genes associated with pre-adipocyte differentiation in YIMAs compared with control cells (Fig. 5L). BODIPY and Oil Red O staining confirmed that knockdown of SRSF3 promoted lipid droplet formation (Fig. 5M, N). These results imply that SRSF3 contributes to the suppression of proliferation and the inhibition of differentiation in YIMAs. Owing to the insufficient overexpression efficiency of SRSF3, further experiments were not conducted.

### *hnRNP A1 facilitates the nuclear egress of circCDK14 via m6A methylation*

**Fig. 6:**
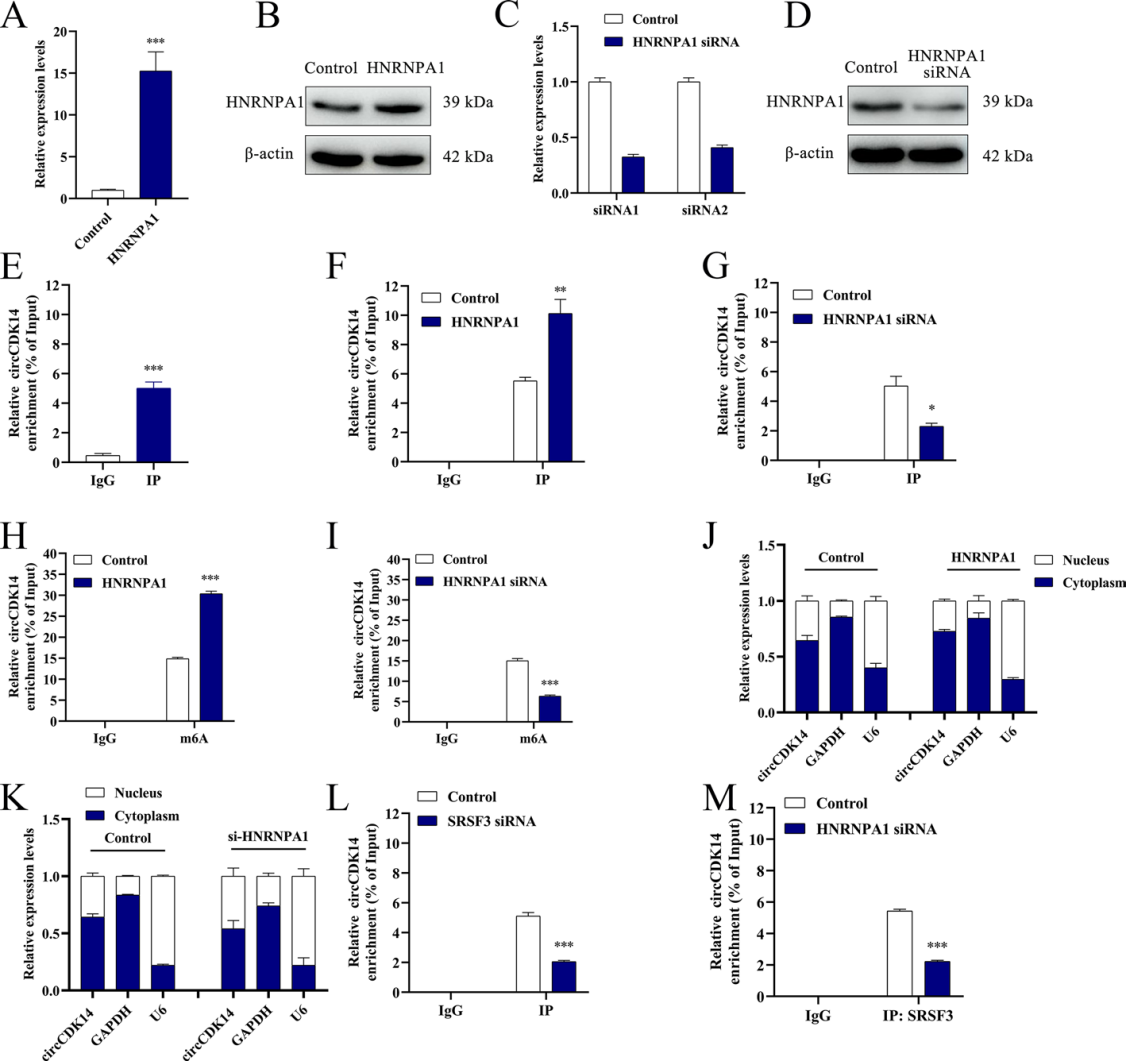
hnRNP A1 promotes nuclear export of m6A-modified circCDK14. **A** and **B** The hnRNP A1 mRNA (**A**) and protein (**B**) expression level after hnRNP A1 overexpression; **C** and **D** the hnRNP A1 mRNA (**C**) and protein (**D**) expression level after hnRNP A1 interference; **E** hnRNP A1 antibody for circCDK14 enrichment; **F**–**G** hnRNP A1 antibody for circCDK14 enrichment after hnRNP A1 overexpression or interference; **H**–**I** m6A antibody for circCDK14 enrichment after hnRNP A1 overexpression or interference; **J**–**K** circCDK14 expression in the nucleus and cytoplasm after hnRNP A1 overexpression or interference; **L** the binding relationship between hnRNP A1 and circCDK14 after SRSF3 interference; **M** the binding relationship between SRSF3 and circCDK14 after hnRNP A1 interference. The western blot, RIP and MeRIP were repeated 3 times (*n* = 3) and other experiments were performed in triplicate and repeated 3 times (*n* = 9)

Since hnRNP A1 and SRSF3 may interact during RNA splicing in cells, we further explored the regulatory mechanisms of hnRNP A1 and SRSF3 in circCDK14 m6A modification. qRT-PCR data revealed that overexpression of hnRNP A1 resulted in a marked elevation in its expression levels compared with control cells. Conversely, the application of hnRNP A1 siRNA1 significantly diminished its expression (Fig. 6A–D). We further confirmed the binding between hnRNP A1 and circCDK14 using RIP, which was influenced by hnRNP A1 expression levels (Fig. 6E–G). MeRIP results revealed that the upregulation of hnRNP A1 increased circCDK14 m6A levels compared with control cells, whereas downregulation decreased them, indicating the impact of hnRNP A1 on circCDK14 m6A modification (Fig. 6H, I). Nucleoplasmic separation assays demonstrated that circCDK14 was mainly localised in the cytoplasm after hnRNP A1 overexpression; however, knockdown of hnRNP A1 promoted the retention of circCDK14 in the nucleus (Fig. 6J, K). These results suggest that hnRNP A1 binds to circCDK14 and facilitates its nuclear-to-cytoplasmic export in an m6A-dependent manner. More interestingly, RIP analysis revealed that the interaction between hnRNP A1 and circCDK14 was weakened upon SRSF3 knockdown, and the interaction between SRSF3 and circCDK14 was hindered by hnRNP A1 knockdown (Fig. 6L, M). These findings indicated a synergistic role for SRSF3 and hnRNP A1 in the regulation of circCDK14 m6A modifications.

### hnRNP A1 inhibits proliferation and differentiation of YIMAs

**Fig. 7:**
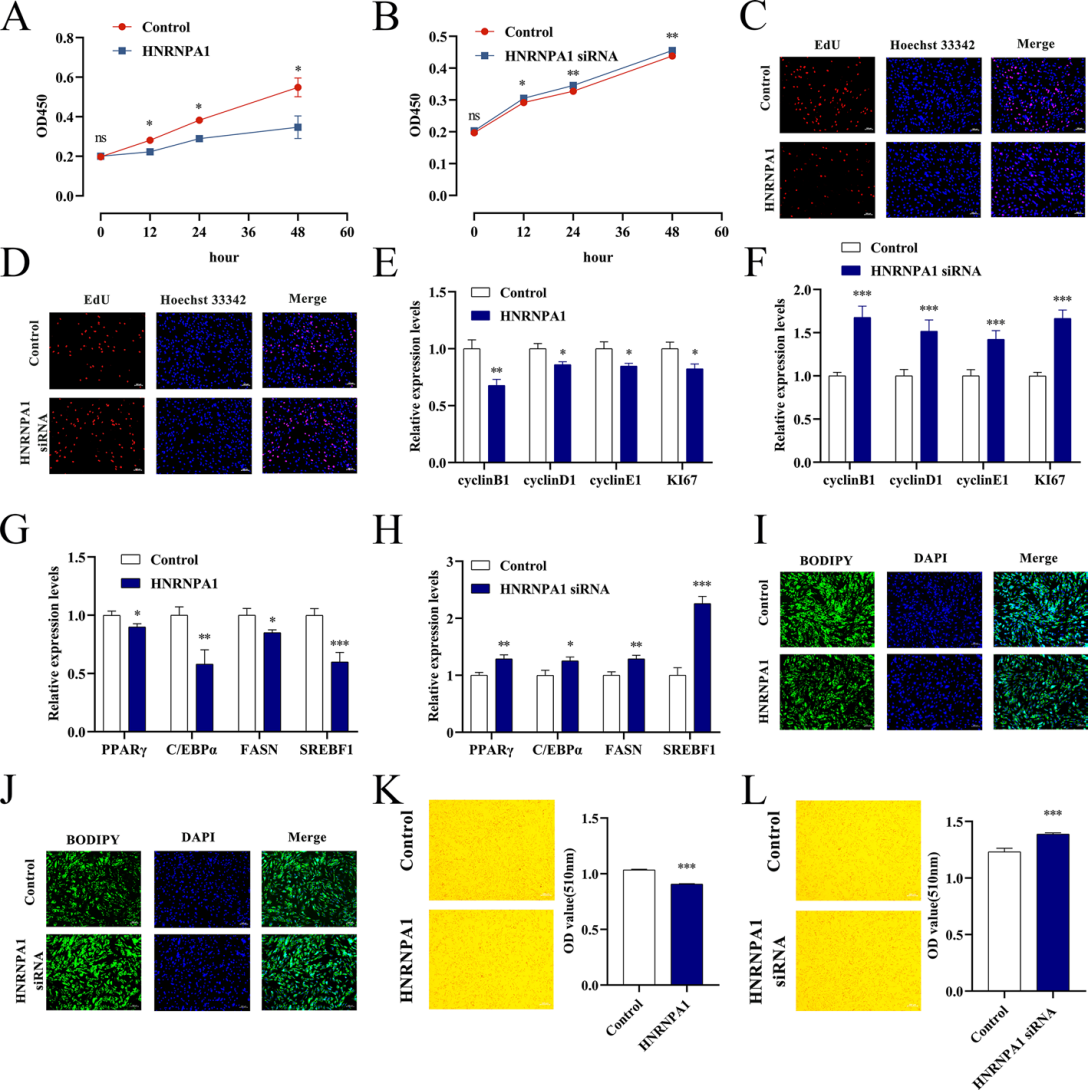
Effects of hnRNP A1 on the proliferation and differentiation of YIMAs. **A**–**B** CCK-8 assay after hnRNP A1 overexpression or interference; **C**–**D** EdU staining after hnRNP A1 overexpression or interference; **E**–**F** proliferation marker gene expression after hnRNP A1 overexpression or interference; **G**–**H** differentiation marker gene expression after hnRNP A1 overexpression or interference; **I**–**J** BODIPY staining after hnRNP A1 overexpression or interference; **K**–**L** Oil Red O staining after hnRNP A1 overexpression or interference. The CCK-8 assay was repeated 6 times (*n* = 6), and the other experiments were performed in triplicate and repeated 3 times (*n* = 9)

Subsequent investigations revealed the effect of hnRNP A1 on fat deposition in yaks. CCK-8 and EdU assays revealed that upregulation of hnRNP A1 led to a significant decrease in cell proliferation capacity and EdU binding in YIMAs compared with control cells, whereas knockdown of hnRNP A1 resulted in enhanced cell proliferation capacity (Fig. 7A–D). Furthermore, qRT-PCR showed that overexpression of hnRNP A1 inhibited the expression of proliferation marker genes in yaks compared with control cells, whereas knockdown of hnRNP A1 had the opposite effect (Fig. 7E, F). Moreover, hnRNP A1 overexpression significantly suppressed the mRNA expression of differentiation marker genes in yaks compared with control cells, with knockdown yielding contrasting results (Fig. 7G, H). BODIPY and Oil Red O staining further demonstrated that hnRNP A1 hindered lipid droplet formation in yaks compared with control cells (Fig. 7I, J). Collectively, these findings indicate that hnRNP A1 plays a critical role in impeding the proliferation and differentiation of YIMAs.

## Discussion

Meat quality, a crucial aspect of the eating experience, is closely linked to the presence of IMF [36]. Studies have identified several factors that influence IMF accumulation, including non-coding RNAs, DNA methylation and m6A modifications [37, 38]. Among these, circRNAs, a type of noncoding RNA that is gaining popularity in research, primarily act as miRNA sponges during fat deposition [14]. Studies have shown that circCWC22 acts as a sponge for miR-3059-x, indirectly regulating the expression of *HMGCL*, thereby affecting IMF deposition in yaks [15]. Moreover, circFUT10 plays a crucial role in the proliferation and differentiation of adipocytes by competitively binding to miRNAs belonging to the let-7 family [13, 39]. These findings reveal the significant role of circRNAs in regulating fat deposition in animals. While research on circRNAs and fat development has mainly focused on humans, pigs, cattle and sheep [40–43], investigations into their role in yak IMF are still in the early stages. In the present study, we observed dynamic changes in circCDK14 expression at different stages of differentiation, which correlated with IMF deposition in yaks. The manipulation of circCDK14 levels through knockdown and overexpression experiments revealed its effect on IMF deposition in yaks. However, the role of circCDK14 in cell proliferation and differentiation to be relatively limited. This can be attributed to two main factors. First, IMF content is a quantitative trait regulated by a complex network involving multiple genetic loci, including genes and non-coding RNAs related to proliferation and differentiation. Second, circRNAs, as a type of non-coding RNA, generally play a fine-tuning role during lipid deposition. The further in-depth exploration of the mechanism of circCDK14 during cell proliferation and differentiation will be our next work. Exon-derived circRNAs are typically located in the cytoplasm where they bind competitively to miRNAs, thereby relieving miRNA-mediated suppression of their target mRNAs [39]. Our findings, supported by the nucleoplasmic separation data, indicate that circCDK14 is predominantly localised in the cytoplasm. In addition, dual-luciferase and functional experiments further indicated that cirCDK14 could act as an miR-4492-z sponge to affect IMF deposition in yaks. Meanwhile, through ceRNA prediction analysis, we identified that circCDK14, miR-4492-z and *FBXO17* might constitute a regulatory axis. However, the results of the dual-luciferase validation experiment showed that miR-4492-z could not effectively bind to *FBXO17*. This finding led us to speculate that there might be a direct or indirect regulatory relationship between circCDK14 and *FBXO17* independent of miR-4492-z. We acknowledge that to fully understand the biological significance of these changes, future studies should be conducted under a broader range of experimental conditions or verified in different cell types. We plan to explore these factors in subsequent research to further validate our findings. In summary, this study contributes to our understanding of yak lipid development and enhances our knowledge of the regulation of cell differentiation in livestock.

Further experiments revealed a strong binding relationship between SRSF3 and circCDK14. SRSF3 functions as a splicing factor, with existing research primarily focusing on its role in the splicing of mRNAs or ncRNAs. Peng et al. [44] demonstrated that SRSF3 influences brown adipocyte development through its involvement in MAP4K4 splicing. In addition, Wang et al. [21] showed that SRSF3 enhances pancreatic cancer resistance to gemcitabine by controlling ANRIL splicing and its association with m6A methylation levels in the lncRNA ANRIL. This highlights the significant regulatory function of SRSF3 in m6A methylation. However, its role in m6A-modified circRNAs remains largely unexplored. Reversible m6A modification, facilitated by methyltransferases, demethylases and m6A-binding proteins, is prevalent in both mRNA and ncRNAs [45–47]. It has been demonstrated that m6A modification contributes to circRNA stability and function in the cytoplasm [48]. We predicted the m6A site of circCDK14 using the SRAMP website and confirmed the presence of m6A modifications through MeRIP experiments. We predicted that the binding site of circCDK14 to SRSF3 is an m6A methylation site. RIP analysis demonstrated a binding relationship between SRSF3 and circCDK14. Moreover, MeRIP experiments indicated a regulatory link between SRSF3 expression and m6A modification of circCDK14. Previous studies have demonstrated that the m6A-writer protein METTL3 interacts with SRSF3 [21]. In alignment with these findings, our study reveals that METTL3 regulates the circCDK14 exit from the nucleus by influencing the m6A expression level of circCDK14. Furthermore, METTL3 rescues the increase in circCDK14 m6A methylation level and nuclear content caused by the decrease in SRSF3 expression. This suggests a potential role of SRSF3 in mediating the nuclear export of circCDK14 through its influence on m6A modification, which ultimately affects yak IMF deposition.

hnRNP A1 was among the RBPs predicted to bind highly to circCDK14. Kumar et al. [49] demonstrated that hnRNP A1 can act as an m6A reader by recognising m6A modifications in SARS-CoV-2 RNAs within infected cells. Moreover, research conducted by Tuersun et al. [50] has uncovered a significant positive correlation between hnRNP A1 and RNA-binding motif protein X-linked, which is recognized as a constituent of the m6A-methylation-binding protein family. This indicates a potential association between hnRNP A1 and m6A modifications. In addition, Kuranaga et al. [28] highlighted the reciprocal relationship between hnRNP A1 and SRSF3 in the splicing of pyruvate kinase muscle 2. It is well known that m6A modifications play important roles in regulating gene expression, including the regulation of splicing events, which, combined with the effect of SRFS3 on circCDK14 m6A methylation, prompted the investigation into whether there is a relationship between hnRNP A1 and SRSF3 in regulating m6A modification. We demonstrated the binding relationship between hnRNP A1 and circCDK14 using RIP analysis, and MeRIP and nucleocytoplasmic separation experiments revealed that hnRNP A1 plays a key role in the methylation and nucleation of circCDK14. Meanwhile, the degree of binding of hnRNP A1 and circCDK14 was weakened after SRSF3 knockdown and vice versa, highlighting the regulatory functions of hnRNP A1 and SRSF3 in circCDK14 m6A modification.

**Fig. 8:**
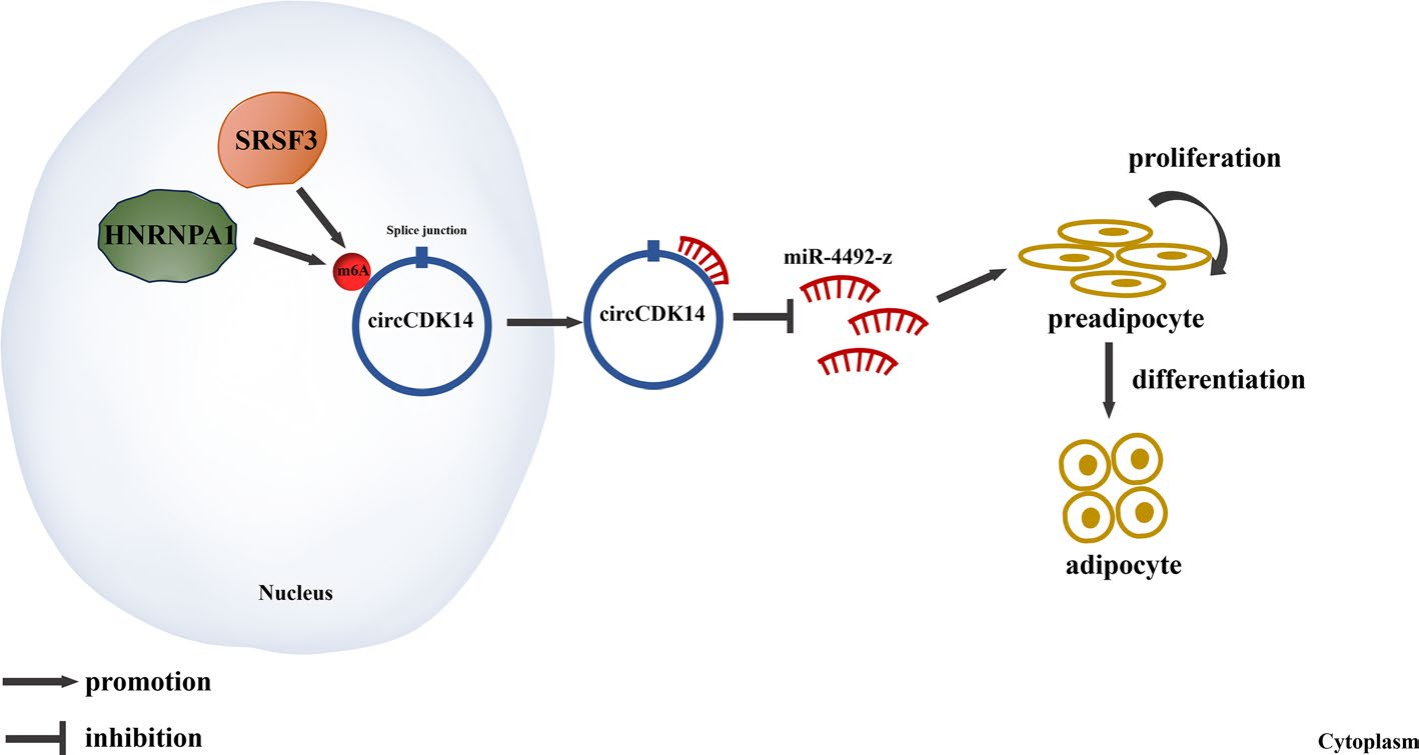
A model for circCDK14 regulation of IMF deposition in yak

In summary, our study identified a novel circRNA, circCDK14, which plays a key role in IMF deposition in yaks. Functionally and mechanistically, circCDK14 regulated IMF deposition by adsorbing miR-4492-z. Furthermore, our research demonstrated that SRSF3 and hnRNP A1 worked together to facilitate the nuclear export of circCDK14 by regulating the m6A modification process, thereby enhancing the biological function of circCDK14 (Fig. 8). These findings offer pioneering insights into the effect of m6A modification on IMF deposition in yaks, opening new avenues for exploring the mechanisms underlying IMF deposition in this species. There are several promising strategies for the practical application of our research findings. First, we propose the development of pharmaceuticals or feed additives designed to enhance the expression of specific circCDK14 molecules. This approach could serve as an effective intervention for stimulating IMF accumulation. Second, by leveraging the circCDK14 molecular marker associated with IMF accumulation, we suggest its use in aiding the selection of livestock with superior meat quality traits. Finally, we recommend employing genetic techniques to identify and select individuals with elevated circCDK14 expression levels, thereby potentially increasing intramuscular fat deposition. These strategic initiatives aim to translate our research into tangible benefits for the livestock industry by enhancing both the quality and economic value of meat production.

## Supplementary Information

Supplementary material 1: Figure 1. METTL3 promotes nuclear export of m6A-modified circCDK14. Table S1: Small RNA details utilized in this study. (A and B) The METTL3 mRNA (A) and protein (B) expression level after METTL3 overexpression; (C and D) The METTL3 mRNA (C) and protein (D) expression level after METTL3 interference; (E-F) METTL3 antibody for circCDK14 enrichment after METTL3 overexpression or interference; (G-H) METTL3 antibody for SRSF3 enrichment after METTL3 overexpression or METTL3 interference; (I-J) m6A antibody for circCDK14 enrichment after METTL3 overexpression or interference; (K) m6A antibody for circCDK14 enrichment after co-transfection of METTL3 interference (METTL3-SI) or control (SI-NC) and wild-type overexpression of METTL3 (OE-WT) or m6A-catalytic defective (OE-MUT) or control (OE-NC); (L) CircCDK14 expression in the nucleus and cytoplasm after co-transfection of METTL3 interference (METTL3-SI) or control (SI-NC) and wild-type overexpression of METTL3; (M) m6A antibody for circCDK14 enrichment after co-transfection of SRSF3 interference (SRSF3-SI) or control (SI-NC) and wild-type overexpression of METTL3 (OE-WT) or m6A-catalytic defective (OE-MUT) or control (OE-NC); (N) CircCDK14 expression in the nucleus and cytoplasm after co-transfection of SRSF3 interference (SRSF3-SI) or control (SI-NC) and wild-type overexpression of METTL3 (OE-WT) or m6A-catalytic defective (OE-MUT) or control (OE-NC). The Western Blot, RIP and MeRIP were repeated 3 times (n = 3), other experiments were performed in triplicate and repeated 3 times (n = 9).

Supplementary material 2: Table S1. Small RNA details utilized in this study. Table S2. Details the primer sequences employed in the study.

## Data Availability

The datasets used and/or analyzed during the current study are available from the corresponding author on reasonable request.

## Abbreviations

IMF: Intramuscular fat
WAT: White adipose tissue
YIMAs: Yak intramuscular pre-adipocytes
circRNAs: Circular RNAs
ceRNA: Competitive endogenous RNAs
SRSF3: Serine/arginine-rich splicing factor 3
hnRNP A1: Nuclear ribonucleoprotein A1
MeRIP: Methylated RNA Immunoprecipitation
CCK-8: Cell counting kit
EdU: 5-Ethynyl-2′-deoxyuridine
RBPs: RNA-binding proteins
BA: Brown adipocyte
RBMX: RNA-binding motif protein X-linked
