# Supplementary figures and images for: SRSF3 and hnRNP A1-mediated m6A-modified circCDK14 regulates intramuscular fat deposition by acting as miR-4492-z sponge
Source: Cell Mol Biol Lett. 2025 Mar 4;30:26. doi: 10.1186/s11658-025-00699-6 (PMC11881307; doi:10.1186/s11658-025-00699-6)

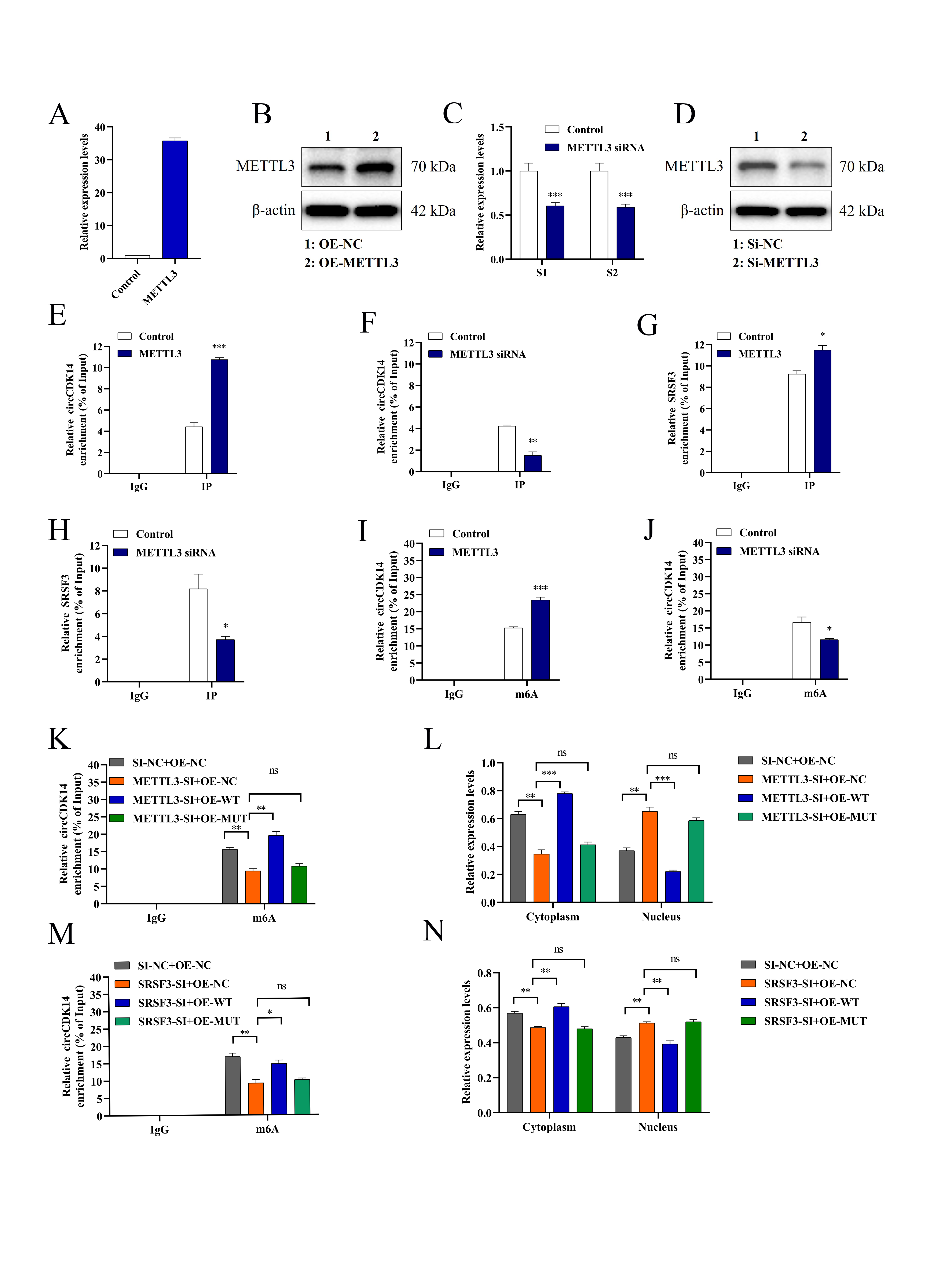

Supplement: Supplementary file 1 — Supplementary material 1: Figure 1. METTL3 promotes nuclear export of m6A-modified circCDK14. Table S1: Small RNA details utilized in this study. (A and B) The METTL3 mRNA (A) and protein (B) expression level after METTL3 overexpression; (C and D) The METTL3 mRNA (C) and protein (D) expression level after METTL3 interference; (E-F) METTL3 antibody for circCDK14 enrichment after METTL3 overexpression or interference; (G-H) METTL3 antibody for SRSF3 enrichment after METTL3 overexpression or METTL3 interference; (I-J) m6A antibody for circCDK14 enrichment after METTL3 overexpression or interference; (K) m6A antibody for circCDK14 enrichment after co-transfection of METTL3 interference (METTL3-SI) or control (SI-NC) and wild-type overexpression of METTL3 (OE-WT) or m6A-catalytic defective (OE-MUT) or control (OE-NC); (L) CircCDK14 expression in the nucleus and cytoplasm after co-transfection of METTL3 interference (METTL3-SI) or control (SI-NC) and wild-type overexpression of METTL3; (M) m6A antibody for circCDK14 enrichment after co-transfection of SRSF3 interference (SRSF3-SI) or control (SI-NC) and wild-type overexpression of METTL3 (OE-WT) or m6A-catalytic defective (OE-MUT) or control (OE-NC); (N) CircCDK14 expression in the nucleus and cytoplasm after co-transfection of SRSF3 interference (SRSF3-SI) or control (SI-NC) and wild-type overexpression of METTL3 (OE-WT) or m6A-catalytic defective (OE-MUT) or control (OE-NC). The Western Blot, RIP and MeRIP were repeated 3 times (n = 3), other experiments were performed in triplicate and repeated 3 times (n = 9). [file 11658_2025_699_MOESM1_ESM.tif]
